# Modulation of biliverdin dynamics and spectral properties by Sandercyanin†

**DOI:** 10.1039/d2ra02880h

**Published:** 2022-07-13

**Authors:** Swagatha Ghosh, Sayan Mondal, Keerti Yadav, Shantanu Aggarwal, Wayne F. Schaefer, Chandrabhas Narayana, Ramaswamy Subramanian

**Affiliations:** Institute for Stem Cell Science and Regenerative Medicine Bangalore 560065 India swagatha.ghosh@gu.se subram68@purdue.edu; Department of Chemistry and Molecular Biology, University of Gothenburg Medicinaregatan 9C 40530 Gothenburg Sweden; Chemistry and Physics of Materials Unit, Jawaharlal Nehru Centre for Advanced Scientific Research Bangalore 560064 Karnataka India; Manipal Academy of Higher Education, Manipal University Madhav Nagar 576104 India; Department of Biological Sciences, University of Wisconsin at Milwaukee Washington County West Bend WI 53095 USA; Department of Biological Sciences, Weldon School of Biomedical Engineering, Purdue University West Lafayette IN 47907 USA

## Abstract

Biliverdin IX-alpha (BV), a tetrapyrrole, is found ubiquitously in most living organisms. It functions as a metabolite, pigment, and signaling compound. While BV is known to bind to diverse protein families such as heme-metabolizing enzymes and phytochromes, not many BV-bound lipocalins (ubiquitous, small lipid-binding proteins) have been studied. The molecular basis of binding and conformational selectivity of BV in lipocalins remains unexplained. Sandercyanin (SFP)–BV complex is a blue lipocalin protein present in the mucus of the Canadian walleye (*Stizostedion vitreum*). In this study, we present the structures and binding modes of BV to SFP. Using a combination of designed site-directed mutations, X-ray crystallography, UV/VIS, and resonance Raman spectroscopy, we have identified multiple conformations of BV that are stabilized in the binding pocket of SFP. In complex with the protein, these conformers generate varied spectroscopic signatures both in their absorption and fluorescence spectra. We show that despite no covalent anchor, structural heterogeneity of the chromophore is primarily driven by the D-ring pyrrole of BV. Our work shows how conformational promiscuity of BV is correlated to the rearrangement of amino acids in the protein matrix leading to modulation of spectral properties.

## Abstract 2

Biliverdin IX-alpha undergoes rotation around the D-ring pyrrole and displays a broad far-red absorbance on binding to monomeric Sandercyanin variant (orange) compared to the wild-type tetrameric protein (cyan).

## Graphical abstract

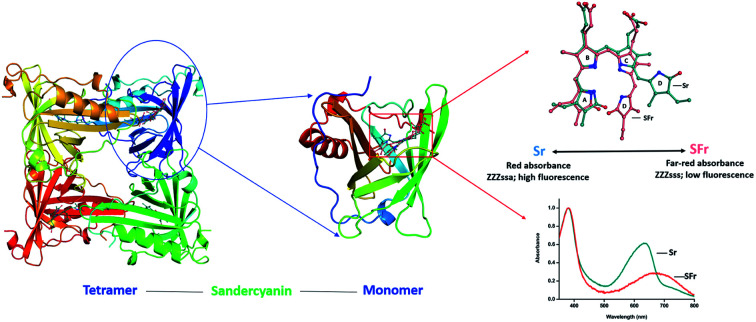

## Introduction

Biliverdin IX-alpha (BV) is a pigment responsible for characteristic blue-green coloration in many living organisms. Along with other tetrapyrroles, BV often acts as a natural chromophore of phytochromes (Phys) in plants, bacteria, fungi, and cyanobacteria. BV regulates various physiological responses, including chlorophyll synthesis, photoperiodic seed germination, flowering, and photo-taxis.^1–4^ BV is a metabolite of heme catabolism. Its presence is used to gain evolutionary and ecological advantages by marine fish,^5^ lizards,^6,7^ chlorotic tree-frogs,^8^ and insects.^9,10^

Sandercyanin (SFP) is a BV-binding lipocalin protein found in the skin mucus of the Canadian walleye, *Stizostedion vitreum* (formerly *Sander vitreus*).^11,12^ We have previously reported the spectral properties and structures of tetrameric wild-type SFP–BV complex.^11,13^ BV-bound SFP shows an intense blue color and absorbances at 380 nm and 630 nm. BV associates non-covalently to each SFP monomer in a ZZZssa conformation and is stabilized by aromatic stacking, hydrophobic interactions, and water-mediated H-bonding inside the lipocalin-like beta-barrel fold of SFP. The binding of BV induces tetramerization of SFP *via* interaction between amino acid residues from adjacent monomeric subunits. The reported protein tetrameric structures suggest that the molecular properties are significantly modified if the protein is engineered into a monomeric form due to the loss of interactions between BV and the neighboring subunits.^11,14^ In other photoactive proteins that use BV as a chromophore (example, bacteriophytochromes (BPh)), the ethylidene side chain of the A-ring of BV is covalently linked to the protein chain *via* a thioether bond with a conserved cysteine residue. BV conformation remains unperturbed in the ground state on changing the native dimeric state of BPh to a monomeric chromophore binding domain.^15,16^ However, BPhs are well-studied systems for light-induced isomerization around the D-ring pyrrole of BV from a dark or red-absorbing state to a far-red absorbing state.^17^

In this report, we elucidate the influence of the oligomeric state of SFP on the conformational selection of BV. Based on the results from the monomeric variants of SFP, we created variants that altered the BV-binding pocket. These variants show interesting differences in their spectral properties compared to the wild-type protein. We use X-ray crystallography to determine structures of these variants and combine it with resonance Raman spectroscopy to provide a molecular explanation for the observed difference in spectral properties.

## Materials and methods

### Site-directed mutagenesis, protein expression, and purification of SFP variants

The gene for wild-type SFP cloned into pET21a(+) was used as a template for generating site-specific mutations. We performed a whole-vector polymerase chain reaction (PCR) of the template gene using high-fidelity Phusion DNA polymerase (New England Biolabs). The presence of the mutation was confirmed by sequencing the plasmid. Expression and purification of SFP variants were performed using methods published for the wild-type protein.^11^ All proteins were purified by size-exclusion chromatography (SEC) using a Superdex 200 10/300 GL column (Merck). BV-binding to SFP variants is confirmed by monitoring absorbance at 380 nm and 630 nm in the SEC profiles. For monomeric and tetrameric variants, blue-colored BV-bound fractions corresponding to the size of ∼18 kDa and ∼75 kDa, respectively, were identified by using a standard protein mixture (BioRAD gel filtration standard #1511901) used for calibration of the SEC column. Purified fractions were collected and used for spectroscopy and crystallographic studies. Apo-protein of each variant was purified using the same method except for the addition of BV.

### Absorbance and fluorescence spectroscopy

All experiments were performed with freshly purified protein samples (apo and BV-bound forms) at pH 7.5 and room temperature. UV-visible absorbance spectra of SFP variants were recorded on an Ultraspec 2100 pro spectrophotometer from Amersham Biosciences. Fluorescence and binding studies of SFP variants were monitored on Horiba Jobin Yvon Fluoromax-4 fluorimeter as described in the previous report.^11^ Briefly, the apoprotein of SFP was titrated with different concentrations of BV, and a change in fluorescence intensity was recorded on excitation at 380 nm and 630 nm. The emission values (at peak maxima) from both excitations were combined and analyzed by GraphPad Prism 9.4.0 to get a tentative estimate of the dissociation constant (*K*_d_). OriginPro 8 software was used for plotting of binding curve. The equation for *K*_d_ calculation is the same as described in our previous paper.^11^

### Protein crystallization, data collection, and structural analysis

Purified SFP variants were mixed with excess BV before crystallization. Hanging drops were set up at 4 °C using a Mosquito high-throughput crystallization system from TTP life sciences, and crystals were grown by vapor diffusion. V71E protein bound to BV (11 mg mL^−1^) crystallized in 0.2 M calcium chloride dihydrate, 20% PEG 3350 at pH 5.1. The L135E protein bound to BV (26 mg mL^−1^) crystallized with 10% PEG 1000 and 10% PEG 8000 as precipitants. The Y142A protein bound to BV (9 mg mL^−1^) crystallized in 0.1 M Bis-Tris pH 5.5 and 3 M NaCl as the precipitant. The BV-bound protein crystals obtained in various conditions were flash-cooled in liquid nitrogen after soaking in 10% ethylene glycol as a cryo-protectant. Crystallography datasets of L135E, V71E, and Y142A SFP variants were collected at BM14, ID23-2, and ID30A-1 beamlines at ESRF (Grenoble, France). The images were indexed, integrated, and scaled using HKL2000 (ref. 18)/d*TREK^19^/MOSFLM.^20^ Structures were determined by molecular replacement method using Phaser^21^ and the wild-type SFP structure (PDB ID: 5EZ2) as starting model. All structures were refined with Refmac^22^ and PHENIX.^23^ Model building was carried out using the program Coot.^24^ All structural illustrations were generated using PyMOL.^25^ All parameters of data collection and refinement statistics are summarized in Table S1.† All the coordinates and structure factors have been deposited in the PDB.

### Resonance Raman spectroscopy

Resonance Raman (RR) spectra of BV-bound wild-type SFP and Y142A variants were recorded using LabRAM HR Evolution confocal Raman microscope (HORIBA Jobin Yvon S.A.S., France) equipped with a spectrograph of 800 mm focal length and a Peltier-cooled CCD to register the spectra. A holographic diffraction grating with 1800 gr per mm, and a 300 μm spectrograph entrance slit was used for high spectral resolution. All protein samples (250 μM concentration) were prepared in phosphate buffer at pH 7.4. Samples were loaded in EPR quality capillary made of Suprasil quartz of outer diameter of 3 mm (Wilmad-LabGlass, NJ, USA), and RR spectra were recorded in 180° backscattering geometry. Samples were resonantly excited with 405 nm or 532 nm solid-state laser operated in CW mode through a 50× objective with a numerical aperture (NA) of 0.5 (Model 1-U2N350, Olympus GmBh, Germany). The laser power on the sample was 4–5 mW for 405 nm and 8–9 mW for 532 nm wavelength. For the 405 nm excitation, each spectrum was recorded after 90 s exposure and accumulated for 270 s. In the case of 532 nm excitation, an exposure of 20 s and a total accumulation time of 100 s were used to acquire a spectrum. The final spectrum was an average of 3 to 5 spectra, each recorded from a fresh sample. Recorded RR spectra were analyzed using LabSpec 5 and Origin 7.

## Results

### Engineering monomeric variants of SFP bound to BV

**Fig. 1:**
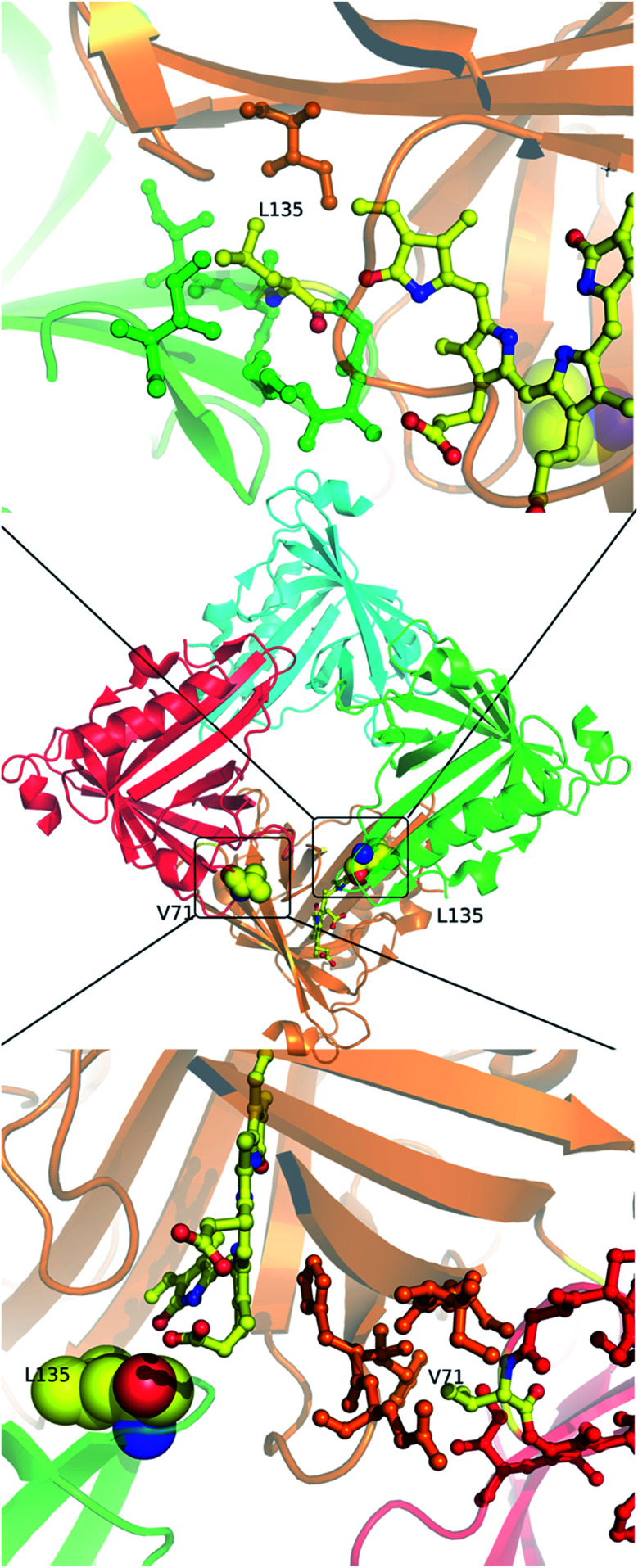
In the center is the tetramer of the published wild-type structure of the SFP–BV complex (PDB 5EZ2). The four monomers are shown in different colors. At the interface of the orange and green subunits (interface 1) is L135 – a zoomed-in view of the interface is shown above. L135 is from the green subunit. At the interface of the orange and the red subunits (interface 2) is V71 – a zoomed view of this interface is shown below. V71 is from the red subunit. The BV in both cases is from the orange subunit. Except for V71, L135, and BV, the residues are colored the same color as their subunits.

**Fig. 2:**
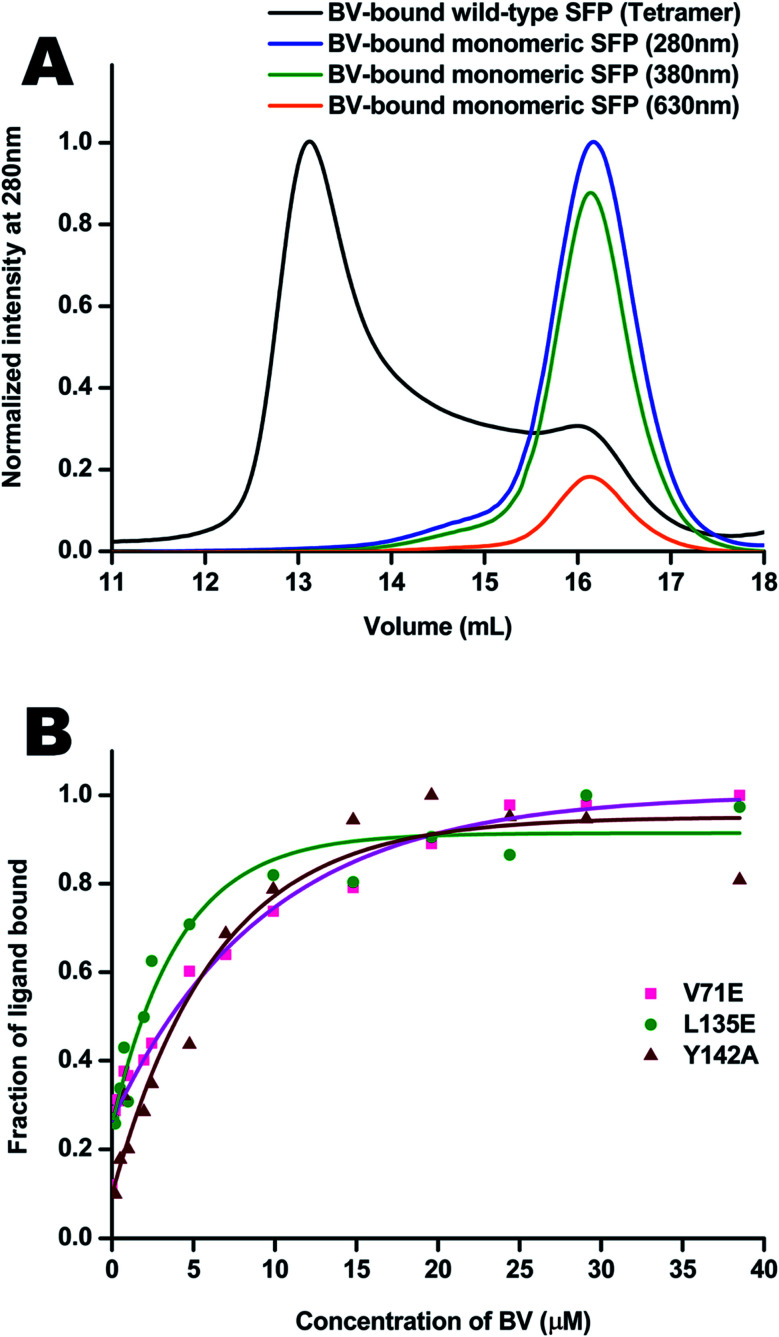
(A) Size-exclusion profiles of the BV-bound wild-type tetrameric protein are black. It elutes at ∼13.4 mL on the S200 analytical column. There is a minor monomeric peak around 16 mL, even in the tetramer. The monomeric forms elute at 16 mL as observed by absorption at 280 nm, 380 nm, and 630 nm, where the protein–BV complexes absorb. (B) Binding curves of BV to the V71E protein in magenta, L135E in green, and Y142A in brown. The *K*_d_ of BV to wild-type protein is 7.06 μM (±0.02) μM. V71E, L135E, and Y142A have *K*_d_ values of 1.97 (±0.12) μM, 1.2 (±0.10) μM, and 4.1 (±0.09) μM, respectively.

Molecular properties and BV-bound structures of wild-type SFP were reported by us earlier.^11,13^ In the wild-type protein, BV binding drives ligand-induced oligomerization from monomeric apoprotein to a BV-bound tetrameric form. Each monomer of the tetramer interacts with two other monomers. We picked residues in two interfaces to create site-directed mutations (Fig. 1). Both are buried hydrophobic residues in the interface of two subunits. We believed that mutating them into charged residues would disrupt the interface and create monomers. Two mutations – L135E (from interface 1) and V71E (from interface 2) stayed monomers on binding to BV (Fig. 2A) as identified by size-exclusion chromatography profiles. The overlap of the 280 nm chromatogram with the 380 nm and the 630 nm profiles confirms that BV is bound to the protein. The affinity of BV to the SFP variants are in similar order of magnitude to the wild-type protein (Fig. 2B). Similar binding affinities of monomer and wild-type protein suggest ligation of BV to SFP is independent of the oligomeric state of the protein. Mutating these hydrophobic residues into polar/charged residues disrupted the binding interface. The purified L135E and V71E variants of SFP (the BV-bound monomeric variants) showed a distinct blue-green coloration (ESI Fig. S1†) under physiological conditions, unlike wild-type protein that appears deep-blue in color.^11^ We characterized the two BV-bound monomeric variants by UV-VIS and fluorescence spectroscopy.

**Fig. 3:**
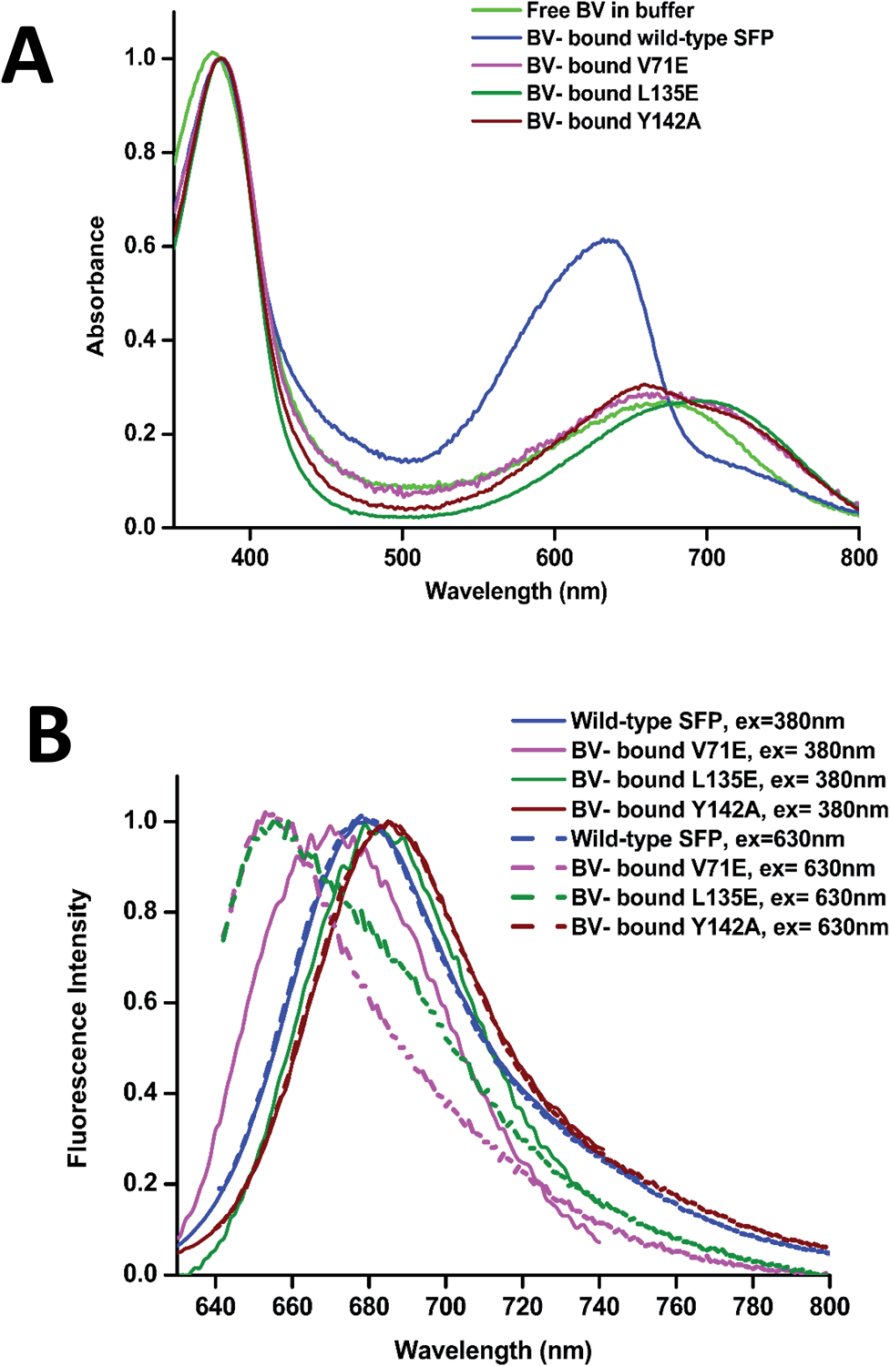
(A) Normalized (at 380 nm) absorption spectra of free BV in light green, wild-type SFP–BV in blue, V71E–BV in magenta, L135E–BV complex in dark green, and Y142A–BV complex in brown. (B) Normalized fluorescence spectra of BV-bound wild-type SFP are blue; V71E in magenta, L135E in green, and Y142A in brown. The continuous lines are the fluorescence emission spectra when excited at 380 nm, while the broken lines are the fluorescence emission spectra when excited at 630 nm. While the fluorescence spectra are similar, there is a significant difference in the far-red region in the absorption spectra of the different variants.

The absorbance spectra of BV-bound V71E and L135E show maxima at 380 nm and an attenuated red-shifted broad peak at the far-red wavelengths (∼680–700 nm) compared to the wild-type protein, where the red-absorbance is at 630 nm (Fig. 3A). The absorbance in the far-red wavelength resembles that of free BV, indicating that the loss of oligomerization enhances exchange between bound and free BV due to its non-covalent nature of interaction with SFP. In BV-binding BPhs, a red shift in absorbance has been associated with the flipping of the D-ring through a *trans*–*cis* isomerization of the double bond (C15=C16) that connects ring C to ring D.^26,27^ This would suggest that, for BV-bound monomeric SFP variants, despite the conformational changes in the D-ring, the interactions of the BV to the protein are still preserved. We noticed that these variants show fluorescence on BV-binding. Excitation of these monomeric variants at 380 nm and 630 nm yields red fluorescence with maxima between 650 nm and 680 nm for different variants (Fig. 3B). A broad absorbance spectrum near the red wavelengths could indicate the presence of multiple conformers of BV inside these monomeric variants, which possibly contribute differently to the red emission on excitation at 380 nm and 630 nm. We also report a significantly diminished fluorescence quantum yield compared to the wild-type protein. The fluorescence quantum yield of the wild-type protein is 0.016. The V71E and L135E mutations showed an order of magnitude reduction, with fluorescence quantum yields of 0.003 and 0.0025, respectively (for excitation at 630 nm). The decrease in quantum yield was not surprising as we had predicted that BV would be more exposed to the aqueous environment in the monomers, providing paths for non-radiative loss of energy.^11,28,29^ Unlike wild-type SFP complex, which binds to a specific conformation of BV, decreased fluorescence quantum yield of monomeric complexes (measured at a specific wavelength) could be due to the presence of several conformers of BV showing variable fluorescence at different excitation wavelengths. The best method to elucidate the molecular basis of the observed spectral changes is by determining the structure of the V71E and L135E variants of the protein in complex with BV by X-ray crystallography.

### Structures of BV-bound V71E and L135E variants

**Fig. 4:**
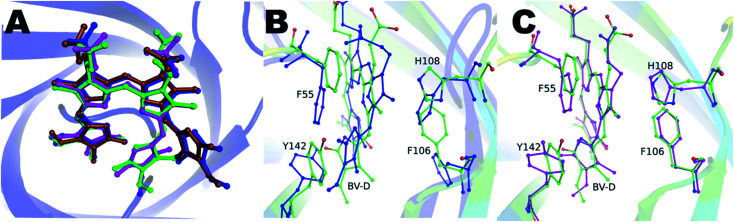
(A) After superposition of the protein structures, the BV conformation is depicted as bound to the wild-type (blue), V71E (magenta), L135E (green), and Y142A (brown) variants. The D-ring is rotated around the C14–C15 bond by 122° and 125° in V71E and L135E, respectively. (B) In blue are the BV and the side chains orientations of aromatic residues of the wild-type protein, and in green are the residues from the L135E structure. The figure clearly illustrates the change in conformation of F55, Y142, F106, and H108 resulting from the D-ring rotation. (C) Comparison of the BV and the same side chains between V71E (magenta) and L135E (green). The reorientation of the side chains is independent of the mutation but only depends on the conformation of the D-ring of BV.

Blue-green crystals of the proteins were obtained after a few days at 4 °C (ESI Fig. S1†). The BV-bound structures of V71E and L135E were determined at 2.5 Å and 2.75 Å resolution, respectively, using wild-type protein structure (PDB ID: 5EZ2) as a model for molecular replacement. Data collection and refinement statistics of BV-bound structures of V71E and L135E are summarized in Table S1.† The crystal packing interactions of BV-bound monomeric variants do not show enough buried surface area to suggest oligomerization. The overall fold of the monomeric variant proteins is very similar to the wild-type protein, with BV bound at the center of the barrel. Loss of oligomerization created a vacant space in the monomer near the open/wider end of the barrel, exposing BV to an aqueous environment at the site of dimerization. N-terminal amino acids and protein backbone of loop residues (L50–E58, R80–I86, H108–V114, and I133–H139) show movement (ESI Fig. S2†). In all the refined structures, the electron density for the BV was well resolved, and the BV was modeled (ESI Fig. S3A and B†). Both structures show one pre-dominant conformation of BV, in spite of a broad absorbance spectrum at the red wavelengths. In the BV-binding pocket, both monomeric variants revealed a rotated D-ring pyrrole of BV around the C14–C15 single bond. Measured torsion angles were 122° and 125° (counter-clockwise) for V71E and L135E, respectively. The conformation of BV is ZZZsss in the variants compared to ZZZssa in wild-type SFP (Fig. 4A). The B and C rings show slight deviation from the native (ZZZssa) state, but the A-ring remains unperturbed even though there is no covalent linkage between the protein and BV like in the phytochromes.^30^ Relative B-factors of the different tetrapyrrole rings with respect to each other are a good indicator of the mobility of the different rings in the structure. The tetrapyrrole rings of the BV in the wild-type are all well-ordered and stabilized by interactions with the protein residues. However, the atoms of the D-ring of the L135E have larger B-factors suggesting that even in the crystal structure, this ring is more flexible. Light-induced isomerization of the C15=C16 double bond of BV has been proposed in the Bphs.^26^ In the SFP–BV complex, the conformational changes in BV seem to be due to the rotation around the single bond C14–C15. This rotation suggests flexibility in the D-ring position in the ground state rather than a specific state induced by irradiation.

Aromatic residues F55, F106, H108, and Y142, undergo concerted rearrangement of side chains to stabilize the interaction with BV in the protein (Fig. 4B and C). In the wild-type protein, the orientation of the D-ring is stabilized by interactions with residues in the neighboring subunit. However, in the monomeric forms, the orientation of the D-ring of BV is stabilized by interactions with the A-ring of BV (the D-ring moves closer to the A-ring of BV) and stacking interactions with the Y142 side chain (which rotates to stack with the D-ring). The changes are concerted on both sides of the BV, with F55 and Y142 on one side and F106 and H108 on the other. In the V71E structure, water molecules occupy the original space vacated by the reoriented side-chains of F55 and Y142. The lower resolution structure of L135E does not allow modeling of structured waters replacing the rotated residues; however, empty space possibly suggests randomly-oriented or the presence of low occupancy water molecules. In both structures, non-aromatic amino acids (such as D47 and Q56) near BV show subtle structural changes. The C-pyrrole of BV and its propionic chain get displaced with the overall chromophore moving slightly upwards from BV bound to the wild-type protein (Fig. 4A). Interestingly, with similar conformational freedom to the D-ring of BV, the non-covalently linked A-ring remains unperturbed and stabilized by surrounding amino acid residues: D47, A61, A63, Y65, N77, Y116, S144, and V146.

We infer from crystal structures of wild-type and monomeric variants that SFP can accommodate several ground state conformations of BV in the binding pocket. Further, we identified F55, F106, H108, and Y142 as crucial residues in stabilizing BV in ZZZsss conformation in the monomer mutants. A systematic mutation of these residues in the wild-type protein would possibly lead to increased conformational flexibility of BV in the binding pocket. We generated variants of all four amino acids, F55, F106, H108, and Y142, by mutating them into alanine to understand their role in the conformational flexibility of BV bound to SFP.

### The tetrameric Tyr-142-Ala variant

We cloned, expressed, purified, and characterized the spectroscopic properties of the F55A, F106A, H108A, and Y142A variants in complex with BV. All variants are tetrameric on binding to BV (ESI Fig. 4A†). Recently published structure and properties of tetrameric F55A–BV complex revealed a rotated D-ring with a far-red absorbance and no observable fluorescence.^14^ However, F106A, H108A, and Y142A showed fluorescence spectra with slightly shifted peak maxima compared to the wild-type protein (Fig. 3B and ESI Fig. 4B†). Spectral properties of the Y142A–BV complex showed an absorbance maximum at 380 nm and a shift in absorption peak from 630 nm (in wild-type protein) to 660 nm, with a concomitant appearance of the 720 nm far-red band with similar intensity (Fig. 3A and ESI Fig. 4C†). On excitation with 380 nm and 630 nm, Y142A showed a red-shifted fluorescence with maxima at 685 nm and a fluorescence quantum yield (0.004) equivalent to the monomeric SFP variants (Fig. 3B).

We hypothesized that the mutation of the large Tyr into Ala created enough space for the D-ring to rotate in the tetrameric protein giving rise to dual peaks in the red region of the absorbance spectrum. To test this, we determined the crystal structure of the Y142A–BV complex at 2.65 Å (ESI Table S1†). However, to our surprise, the BV adopted the ZZZssa conformation in the crystal structure (Fig. 4A), with a water molecule accommodated in the position of the mutated side chain of Y142 (similar to the V71E structure). The similarity of BV conformation to the wild-type protein could not explain the observed differences in the absorption spectra. To test if conformational heterogeneity did exist in the solution state producing the co-existing red (∼660 nm) and far-red (∼720 nm) absorbing states of Y142A, we decided to probe the wild-type and the Y142A variant using solution-state resonance Raman spectroscopy.

### Conformational plasticity of BV in SFP studied by resonance Raman spectroscopy

**Fig. 5:**
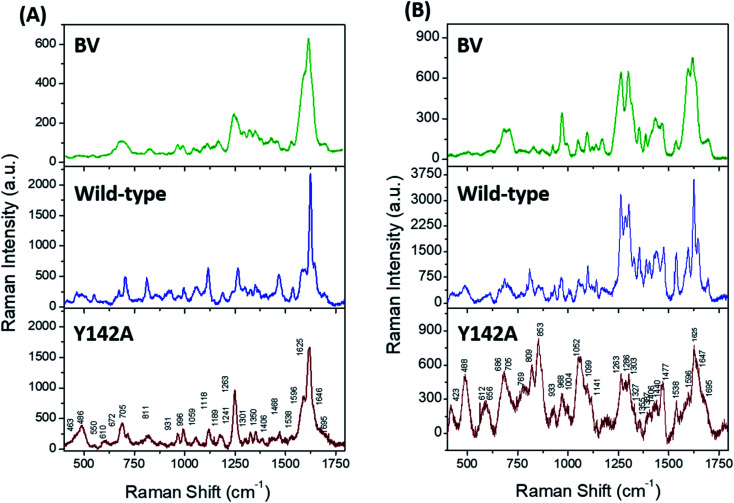
Resonance Raman (RR) spectra of free BV (green), wild-type Sandercyanin–BV complex (blue), and Y142A–BV complex (brown) with (A) 405 nm and (B) 532 nm excitation. Peak broadening around 1600 cm^−1^ suggests the existence of a conformationally flexible D-ring in the solution state of Y142A.

Solution state resonance Raman (RR) spectroscopy near the two absorbance states of the wild-type SFP–BV complex (Fig. 3A, blue) allowed us to probe conformations of BV in SFP.^31,32^ The RR spectra of free- and bound BV to wild-type protein at 405 nm (Fig. 5A) and 532 nm (Fig. 5B) reveal that binding to SFP leads to spectral narrowing of vibrational bands of BV in the high-frequency region. The high-frequency region primarily represents the resonance-enhanced in-plane vibrations coupled to D-ring (Table S2† for normal mode assignments of all bands).^33^ The loss of RR intensity in these bands implies a sudden decrease in polarizability of these modes – probably due to the disruption of the pi–electron conjugation, and suggests possible rotation of the D-ring of BV and stabilization of a specific BV conformation inside SFP.^34^ In contrast to the wild-type protein, BV bound to Y142A shows loss of fine structures of several RR bands in 532 nm excited spectra, specifically four bands at 1695, 1647, 1625, and 1596 cm^−1^ associated with D-ring in-plane stretching vibrations (Fig. 5). Significant broadening of these RR bands strongly suggests the occurrence of multiple BV conformers bound to Y142A in the solution state. Also, a significant reduction of RR intensity in the 1200–1750 cm^−1^ region indicates the loss of pi–electron conjugation between adjacent covalently linked rings and the disruption of non-covalent pi–pi interactions with aromatic residues stabilizing BV.

## Discussion

This paper combines spectroscopy (UV-VIS and RR) and X-ray crystallography to elucidate the influence of SFP on the dynamics of BV and its spectral properties. Our data on monomeric SFP and Y142A shows that the D-ring pyrrole of BV exhibits significant flexibility. Furthermore, monomeric SFP variants form a stable complex with BV but show different spectral properties compared to the wild-type protein. Interestingly, the presence of several ground state conformations suggests conformational promiscuity of BV bound to SFP.

Dark (or resting state) ZZZssa conformation of BV of SFP is similar to BPhs that yields a red-absorption (called Pr in BPhs) state ∼630–645 nm.^11,26,35^ However, unlike BPhs, which undergo photo-isomerization to ZZEssa (or PFr) conformation, BV in SFP shows ground state conformers (ZZZsss) arising from single bond rotation around the C14–C15 single bond. This study refers to them as Sr (red) and SFr (far-red) photo-states.^26,35–37^ Light-induced photoconversion is not yet established in SFP. Nevertheless, both mechanisms (isomerization in Bphs and rotation in SFP) are driven primarily by the D-ring of the BV, despite the significant difference in the protein structures. Covalent bonding of A-ring pyrrole to a conserved cysteine residue in BPhs constraints the structural flexibility of the chromophore in the ground and illuminated state.^38^ In SFP, BV associates non-covalently, and the A-ring possesses similar conformational freedom as D-ring, yet it remains invariant in all the SFP variants (Fig. 4A). Comparison with other lipocalins bound to biliverdin isoforms in the PDB (*Pieris brassicae* (PDB: 1BBP) and *Manduca sexta* (PDB: 1Z24)) show many conserved residues interacting with the A-ring equivalent pyrrole that possibly stabilize the A-ring in SFP.

The RR studies of Y142A mutation reveal a flexible D-ring of BV in solution with two small shoulder peaks in the far-red absorbance, yet only one conformation is observed in the crystal structure. We have shown earlier that in SFP, absorbance peaks at 630 nm and 720 nm correspond to ZZZssa and ZZZsss forms of BV, respectively.^11,14^ The peak at 660 nm of Y142A could be an intermediate that consists of one or more conformers of BV overlapping together. Our earlier report showed that even the apo-protein (monomeric in solution) forms a tetramer at high concentration (PDB 5F1E).^11^ The increased stability provided to the ZZZssa conformation of BV by the residues of the neighboring subunit (Fig. 1) may be sufficient to shift the equilibrium towards this conformation in the high concentration of the protein in the crystals of Y142A. Hence, we conclude that both conformations of BV are possible in the Y142A variant in solution, even though only one is observed in the crystal structure.

There are only two well-characterized vertebrate fluorescent proteins (SFP and UnaG^39^). The reported monomeric forms of SFP are the smallest (∼18 kDa) far-red fluorescent proteins of vertebrate origin to date.^11,40^ Structural studies reveal several parameters responsible for reduced fluorescence quantum yield of monomeric SFP–BV complex. Firstly, exposure to the protic environment in the BV-binding pocket (due to increased water molecules) plays a significant role in quenching the fluorescence of chromophores.^11,33,34,41,42^ Crystal structures of BV complexes of V71E and Y142A reveal water molecules displacing side-changes of aromatic residues F55 and Y142 (absent in the Y142A variant), making it polar at the core of the binding pocket. We have earlier reported that increasing hydrophobicity around free BV and deuteration of wild-type SFP increases the fluorescence intensity, possibly due to reduced non-radiative excited-state proton transfer (ESPT) processes.^11^ Our recent discovery emphasizes that hydrophobicity and stacking of F55 with BV is a crucial factor for fluorescence of SFP–BV complex.^14^ Secondly, rotation of the D-ring of BV to adopt multiple ground-state conformations (ZZZssa and ZZZsss) could affect the quantum yield of the SFP–BV complex. Due to the higher flexibility of BV in monomeric protein (also evident by its broad absorbance spectra) and RR studies on Y142A variant with reduced quantum yield, only a fraction of BV conformers (mainly the ZZZssa isoform) may show fluorescence. This is further supported by studies on fluorescent proteins (FPs) developed from BV-binding BPhs that recognized conformational flexibility of BV as an important factor for reduced fluorescence quantum yield.^28,29,43,44^ Similar to BPhs, we could employ structure-based protein engineering techniques on monomeric SFP variants to generate bright monomeric far-red fluorescent proteins for *in vivo* applications. Our studies suggest that increasing the hydrophobicity of the binding site and attaining a sterically-locked ZZZssa conformer of BV would increase the fluorescence quantum yield of the SFP–BV complex.^44,45^ As SFP is a vertebrate protein, there are many opportunities for utilizing it for cellular applications, where BV is produced intrinsically as a metabolite.^16,46,47^

In conclusion, our work shows BV dynamics in the binding pocket of a lipocalin protein and postulates a dominant structural characteristic of the conserved D-ring pyrrole (despite the protein structure and nature of linkage present within) in driving chemistry of BV- or linear-tetrapyrroles binding proteins across diverse species.^48,49^

## Data availability

The atomic coordinates and structure factors for BV-bound SFP variants have been deposited to the Protein Data Bank (www.pdb.org) with PDB IDs: 7O2Y (V71E), 7O3K (L135E), and 7YX1 (Y142A). All remaining data are contained within the article.

## Author contributions

SG and RS planned the research. SG designed and characterized the clones for detailed studies. SG and KY purified protein, performed UV-VIS spectroscopy, protein crystallization, and analyzed crystallography data. SM and SA carried out RR data collection; SM performed detailed data analysis and interpretation of RR data. RS, CN, WFS, and SG supervised the project. SG and SM wrote the manuscripts and received inputs from all authors.

## Conflicts of interest

The authors declare that they have no conflicts of interest with the contents of this article.

## Supplementary Material

RA-012-D2RA02880H-s001
