# Supplementary material for: Modulation of biliverdin dynamics and spectral properties by Sandercyanin
Source: RSC Adv. 2022 Jul 13;12(31):20296–304. doi: 10.1039/d2ra02880h (PMC9277520; doi:10.1039/d2ra02880h)
Supplement: RA-012-D2RA02880H-s001 [file RA-012-D2RA02880H-s001.pdf]

Electronic Supplementary Material (ESI) for RSC Advances.
This journal is © The Royal Society of Chemistry 2022

Modulation of biliverdin dynamics and spectral properties by Sandercyanin

Swagatha Ghosh[a,b,*,‡], Sayan Mondal[c, ‡,†], Keerti Yadav[a,d], Shantanu Aggarwal[c], Wayne F. Schaefer[e], Chandrabhas Narayana[c], Ramaswamy Subramanian[a,f, *]

[a] Institute for Stem Cell Science and Regenerative Medicine, Bangalore, India 560065

[b] Department of Chemistry and Molecular Biology, University of Gothenburg, Medicinaregatan 9C, 40530 Gothenburg, Sweden

[c] Chemistry and Physics of Materials Unit, Jawaharlal Nehru Centre for Advanced Scientific Research, Bangalore 560064, Karnataka, India

[d] Manipal Academy of Higher education, Manipal University, Madhav Nagar, India 576104

[e] Department of Biological Sciences, University of Wisconsin at Milwaukee, Washington County, West Bend, WI 53095, USA

[f] Department of Biological Sciences and Weldon School of Biomedical Engineering, Purdue University, West Lafayette, IN 47907, USA

Table S1. X-ray crystallographic data collection and refinement statistics of monomeric and tetrameric variants of SFP.

Crystal	V71E -BV (monomer)	L135E -BV (monomer)	Y142A-BV (tetramer)
**PDB ID**	**7O2Y**	**7O3K**	**7YX1**
**Data collection**	ID23-2, ESRF	BM14, ESRF	ID30A-1, ESRF
**Space group**	$P4_1$	$P4_1$	$P6_322$
**Unit cell dimensions**	38.5 38.5 117.6	39.7 39.7 118.9	161.3 161.3 82.2
**Resolution range (A^0)**	36.56 - 2.5 (2.59 - 2.5)	39.65 - 2.75 (2.85 - 2.75)	46.57 - 2.65 (2.78 - 2.65)
**Total reflections**	41927 (3399)	16228	125211
**Unique reflections**	6126 (685)	4795 (703)	18721 (1830)
**Multiplicity**	6.8	3.4	6.7
**Completeness (%)**	99.70 (99.35)	99.2 (98.9)	99.5 (99.5)
**Mean I/sigma(I)**	10.4	9.0	5.1
**Rpim**	0.05(0.24)	0.07(0.35)	0.14(0.26)
**Wilson B-factor**	36.55	43.39	14.5
**R-factor**	0.1732	0.2313	0.2296
**R-free**	0.2535	0.2505	0.2759
**Number of atoms:**			
Macromolecules	1281	1271	2580
Ligands	43	43	86
Water	51	15	122
**Protein residues**	166	166	338

**RMS(bonds)**	0.014	0.013	0.010
**RMS(angles)**	1.95	1.96	1.35
**Ramachandran favored (%)**	95.12	90.24	97.31
**Clashscore**	6.17	12.05	6.69
**Average B-factor:**	40.55	40.53	20.33
Macromolecules	40.63	40.44	20.48
Solvent	39.87	39.14	15.54

(Statistics for the highest-resolution shell are shown in parentheses)

Supplementary Figure S1

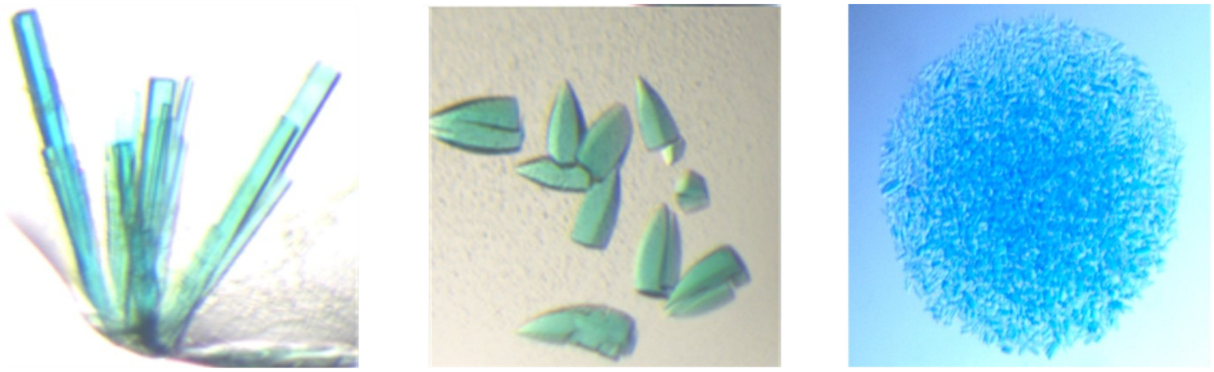

BV- bound Val-71-Glu
11mg/mL
0.2M calcium chloride dihydrate,
20% (w/v) PEG 3350, pH 5.1

BV- bound Leu-135-Glu
26mg/mL
10% (w/v) PEG 1000,
10% (w/v) PEG 8000

BV- bound Tyr-142-Ala
9mg/ml;
0.1M Bis-Tris pH 5.5
3M NaCl

Supplementary Figure S1: Crystals and crystallization conditions of SFP variants in complex with BV. The monomeric variants did not show the same dark blue color as the tetrameric variants. V71E and L135E were monomeric., while Y142A is tetrameric.

Supplementary Figure S2

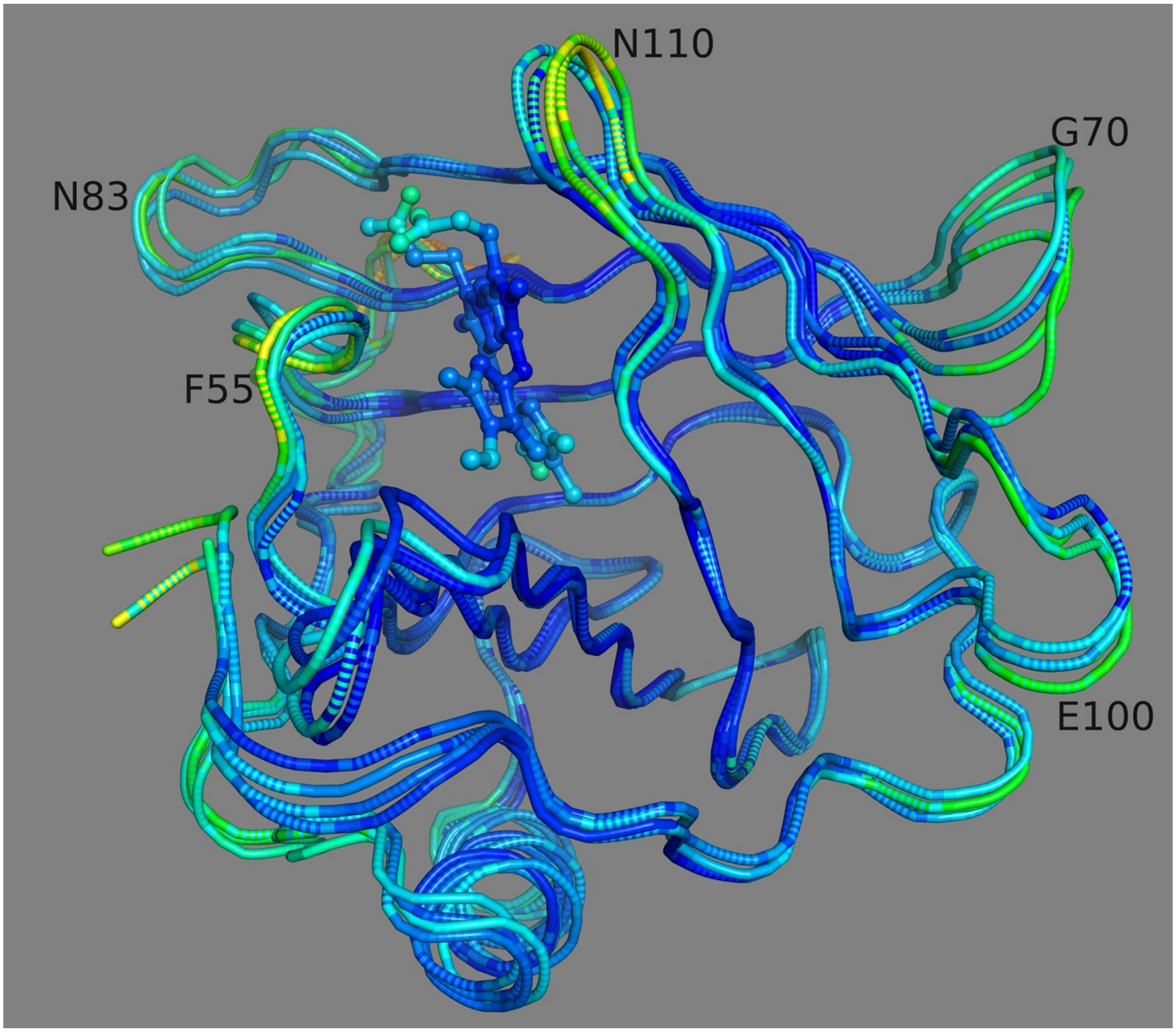

Supplementary Figure S2: Ribbon representation of the superposed structures of wild-type, V71E, L135E and Y142A. The overall structures are the same. The colors of the structures go from blue to red based on B-factor (lower B – blue). There are changes in the loop regions. Biliverdin (BV) binds at the entrance of the binding pocket. In the wild-type tetrameric structure the entrance to the barrel is closed by the neighboring subunit as seen in Figure 1 of the main manuscript. The loops of the monomeric variants are more flexible compared to the loops of the wild-type of Y142A. The BV position of the wild type structure (PDBID 5EZ2) is shown.

Supplementary Figure S3

(A)

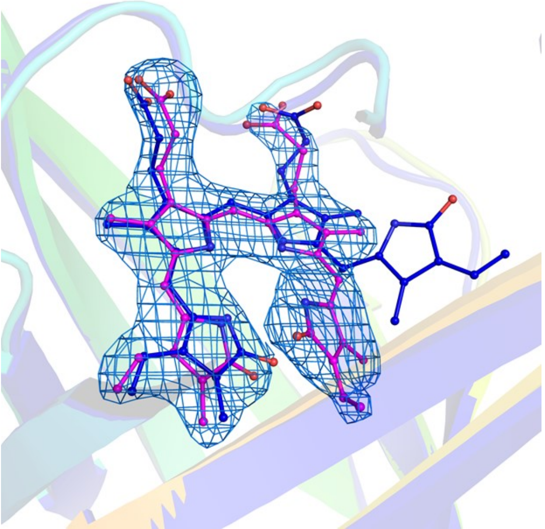

(B)

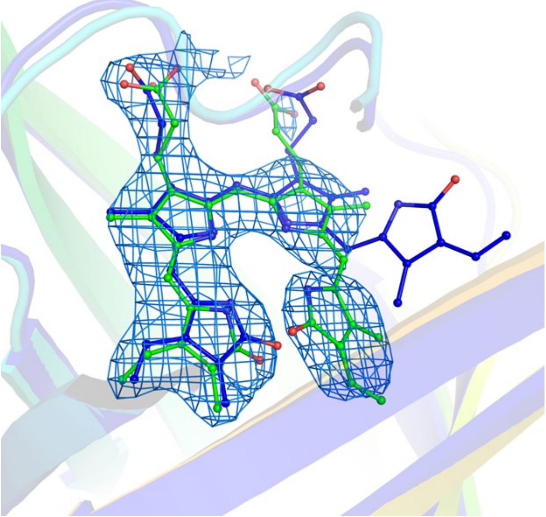

Supplementary Figure 3. BV modelled in the binding pocket of (A) V71E (magenta) and (B) L135E are represented along with 2Fo-Fc maps (contoured at 1σ). The maps clearly show a rotated D-ring in the monomeric variants compared to the wild-type BV (blue).

Supplementary Figure S4

(A)

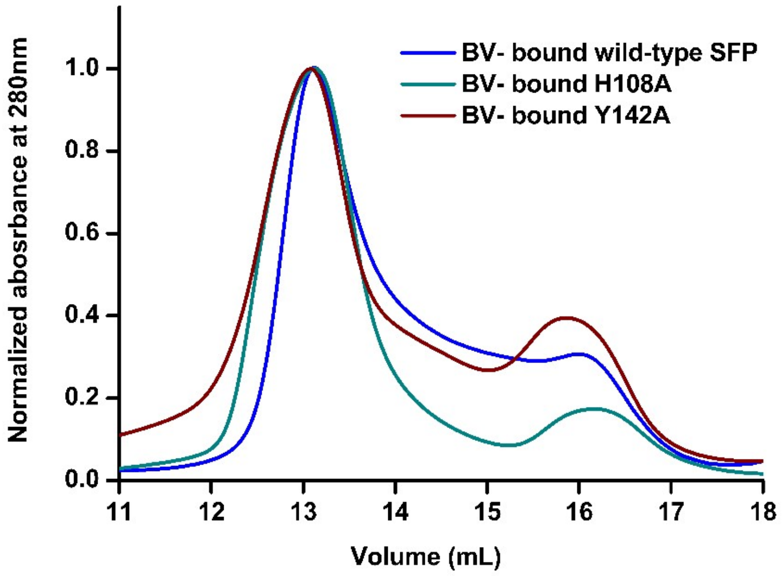

(B)

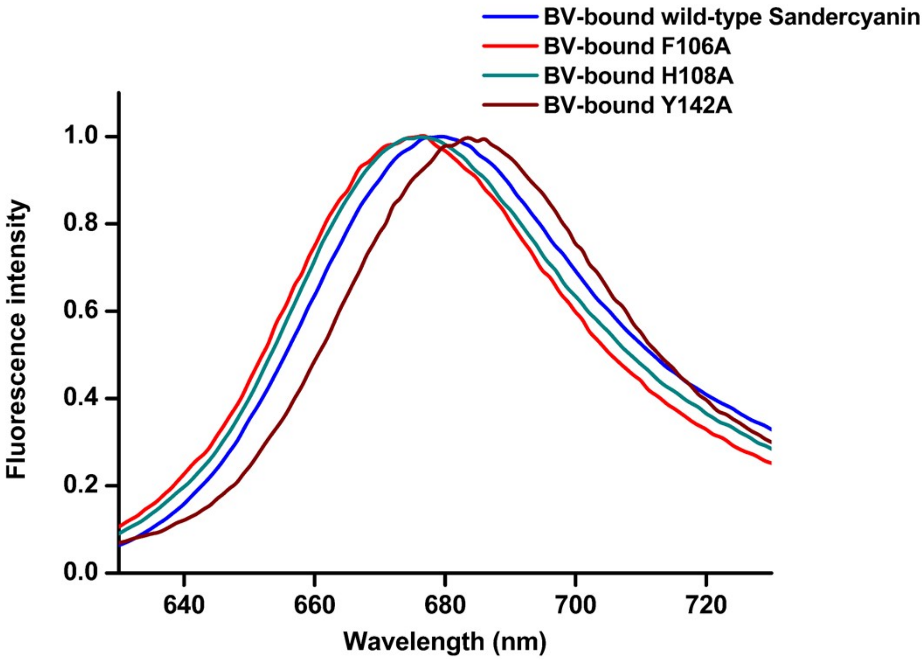

(C)

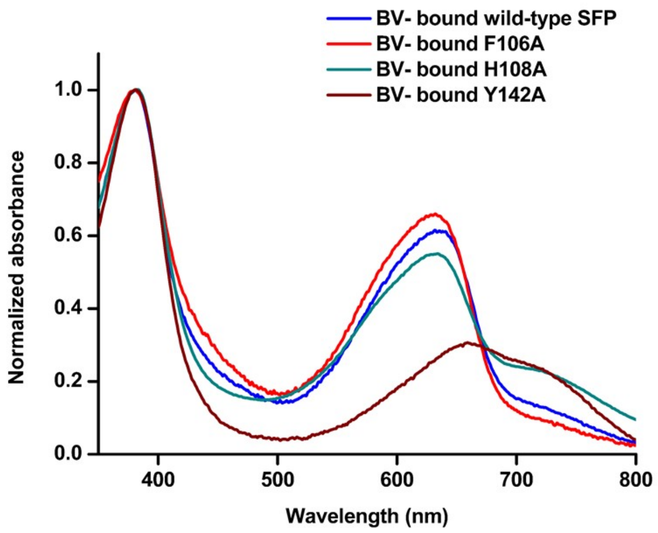

Supplementary Figure 4. (A) Size- exclusion chromatography (SEC) profiles, (B) The Fluorescence emission at excitation 380 nm and (B) absorbance spectra of F106A (red), H108A (deep cyan), Y142A (brown) compared to the wild-type SFP (blue). They have been normalized to 1.0 at the maximum value. Single mutations in the BV-binding site show variation in the spectral properties of Sandercyanin. For SEC, BV- bound solutions of wild-type protein, H108A and Y142A, normalized for the peak corresponding to tetrameric peak at 13.4 mL. The monomeric fractions (~16 mL) are contributed from apo protein. Since BV drives oligomerization in SFP (with apo- protein purely monomeric in solution), a change in concentration of the monomeric fraction (~16mL) among different variants is likely due to difference in the concentration of BV added during purification. F106A has a similar profile (data not shown here) to the wild-type and other tetrameric variants of SFP.

Table S2. Normal mode assignment of resonance Raman bands of free BV, BV-bound wild-type SFP and Y142A-BV complex.

Free BV				SFP-BV complex				Assignment	Localization of normal mode
				Wild type		Y142A mutant			
RR, λ_{exc} = 405 nm	RR, λ_{exc} = 532 nm	Raman, Soln. 1064 nm [1]	SERS, Soln., 514 nm [2]	λ_{exc} = 405 nm	λ_{exc} = 532 nm	λ_{exc} = 405 nm	λ_{exc} = 532 nm		
cm^{-1}	cm^{-1}	cm^{-1}	cm^{-1}	cm^{-1}	cm^{-1}	cm^{-1}	cm^{-1}		
1699, w	1696, vw	1699	-	1695, w	1695, m	1699, w	1693, vw	C=O str.	loc., A-ring, D-ring
-	-	-	-	-	-	-	-	C-C str. ring + C_4=C_5 str.,	loc., A-ring
1636, sh, m	1634, sh, m	-	-	1646, sh, w	1647, sh	1644, sh, m	1648, w	C-C str. ring + C_{15}=C_{16} str. + C-C str. CH-CH_2	loc., D-ring
1616, vs	1618, sh, s	-	1604	1625, vs	1625, vs	1620, vs	1625, m	C-C str. ring + C_{15}=C_{16} str.	loc., D-ring
-	-	-	-	-	-	-	-	C-C str. ring + C_9=C_{10} str.	loc., A-ring, B-ring
1588, sh, s	1591	1593	-	1596, sh, m	1596, m	1591, sh, m	1597, vw	C-C str. ring + C_9=C_{10}-C_{11} str.	loc., A-ring, B-ring
-	-	-	-					C-C str. ring + C-C str. CH-CH_2	loc., D-ring
1528, w	1528, w	-	1544	1538, w	1538, m	1537, vw	1539, vw	in-plane NH be., ring breath.	deloc.
1464, vw	1467	1470	1468	1468, m	1477, m	1471, w	1471, m	C-C str. aliph. + C-N str. ring + CH_3 def.	loc., C-ring
1432, w	1434	-	-		1440, m	1439, vvvw	1440, vvw		-
-	-	-	-	1406, vvw	1406, m	-	1405, vw	CH3 def. + CH be. + ring breath.	loc., C-ring, D-ring
1384, vw	1386	1384	1379	-	1387, m	1386, vvw	1388, vw	CH3 def. + CH be. + C-C str. ring. + NH be.	loc., A-ring
1353, w	1352	-	1352	1350, vw	1355, m	1353, m	1356, vvw	CH3 def. + C-C str. ring + C-C str. aliph.	loc., A-ring, B-ring
1322, w	1321, m, sh	-	-	-	1327, sh, w	1326, m	1327, m	CH be. + CH2 wag. + NH be.	deloc.
1301, vvw	1302, vs	1301	1300	1301, vw	1303, s	1302, vvw	1302, m	NH be. + C-N str. ring + ring breath.	deloc.
-	-	-	-	-	1286, s	-	1285, m	NH be. + CH be.	deloc.
1260, sh, m	1262, vs	-	1260	1263, m	1263, s	1265, m, sh	1263, m	NH be. + ring breath. + CH be.	loc., C-ring, D-ring
1239, m, sh	1244, m, sh	1248	-	1250, sh, vvw	-	1248, s	1244, vvw	NH be. + CH be. + CH2 wag.	deloc.
1224, w, sh	1226, m, sh	-	-	1234, sh, vw	1236, sh, vvw	1232, sh, vw	-	NH be. + CH2 wag.	deloc
-	-	1193	1161	1194, w	-	1188, w	1189, vvw	ring breath. + CH2 wag. + C-C str. aliph.	loc., D-ring
1168, w	1170, w	-	-	-	1168, w	1174	-	ring breath. + CH2 wag.	loc., C-ring
1139, vvw	1138, vvw	-	-	-	1141, m	-	1143, vvw	ring breath. + CH be.	loc., A-ring
1118, vw	1120, vvw	-	-	1118, m	1114, vw	1118, m	1117, vvw	NH be. + ring breath. + CH be. + CH3 def.	deloc.
-	1094, w	1095	1085	1097, sh, vvw	1099, m	1098, vvw	1099, m	C-C str. + C-N str. + NH be. +CH be.	loc., D-ring
-	-	-	-	-	1068, sh	1065, vw	1068, m	CH2 wag. + C-C str. aliph. + CH3 def.	-
1055, vvw	1052, w	-	1042	1059, w	1052, m	1057, m	1052, m	CH3 def.	-

-	-	-	-	-	1014, vw	-	-	CH3 def.	-
-	-	-	-	-	1004, w	-	-	CH2 wag. + ring breath	loc., D-ring
992, w	996, vw, sh	-	993	996, w $^{\$\$}$	994, vvw	993, m	-	CH3 def. + ring breath.	loc., A-ring
966, w	972, m	972	-	-	968, m	966, m	968, vw	CH2 wag. + ring breath. + CH3 def.	loc., C-ring
-	-	-	-	-	956, sh	-	958, sh, vvw	CH2 wag. + ring breath. + CH3 def.	loc., B-ring
923, vvw	926, w	-	936	931, w	933, m	935, vw	933, vw	NH be. + ring breath. + CH be. + $C_9C_{10}C_{11}$ be. + $C_4C_5C_6$ be.	loc., B-ring
-	-	-	909	-	919, vw	-	-	Ring def. + $C_{14}C_{15}C_{16}$ be.	deloc.
-	-	885	-	-	899, w	-	-	ring str.	loc., A-ring
not obs.	873, vvw	-	-	872, vvw	871, w	874, vvw	872, w	CH wag.	-
-	-	835	842	857, vw	853, w	-	852, w	HOOP, C_{15}-H	loc., C_{15}-H
825, w	828, vw	-	-		822, sh	835, sh, vw		Out-of-plane ring def.	loc., D-ring
-	-	818	-	811, m	809, m	820, m	821, w	CH2 wag. + OCO be.	-
-	-	790	-	-	796, sh, vw	-	-	ring breath.	deloc.
-	-	783		-	-	-	-	Out-of-plane ring def.	loc., A-ring
not obs.	761, vvw, *	767	-	-	769, vvw	-	775, vvw	Out-of-plane ring def. + CH2 wag. + C-C str.	loc., A-ring
-	-	722	-	-	722, sh, vw	717, sh, m	720, sh, vw	Out-of-plane ring def. + CH2 wag. + ring str.	loc., B-ring, C-ring
708, w, *	710	717	-	-	711, sh, w	-	708, sh, w	CH2 wag. + ring str	deloc.
-	-	709	-	705, m	700, sh	-	-	out-of-plane NH be. + ring def.	deloc.
-	-	688	-		686, m	688, m	684, vw	in-plane ring def.	deloc.
672, w, *	677	679	680	672, m	673, sh, w	672, sh, vw	-	out-of-plane ring def.	deloc.
647, sh, vw	650, sh, v w	655	642	654, sh, vw	655, m	653, vvw	654, sh, w	In-plane ring def.	deloc.
not obs.	610, vvw	620	-	610, vvw	612, w	609, vw	610, vw	In-plane ring def. + out-of-plane NH be.	-
-	-	-	-	-	581, sh, vw	588, vw	590, vw	In-plane ring def.	deloc.
544, vvvw		-	-	550, m	-	550, vvw	-	ring str. + C-C str. aliph.	loc., A-ring
-	505, vvw	511	-	510, vw	-	508, m	510, sh, w	ring def. + CH2 wag.	loc., B-ring + C-ring
-	-	-	488	486, vw	488, vw	487, m	489, m	out-of-plane ring. def.	loc., A-ring + B-ring
-	-	-	-	463, vvw	464, sh, vw	458, m	-	out-of-plane NH be.	loc., D-ring

[1] ref (1) ; [2] ref (2); abbreviations; s, strong; vs, very strong; m, medium; sh, shoulder; w, weak; vw, very weak; vvw, very very weak; Aliph., aliphatic; str., stretching; be., bending; def., out-of-plane deformation; as., asymmetric; wag., wagging or out-of-plane bend; loc., localized on a ring; breath., in-plane-breathing; $^{\$\$}$ 992 cm-1 band corresponding to A-ring localized mode is used to normalize the 405 nm excited spectrum in Figure 5 panel A.

1. B. Knipp, *et al.*, Regioselective Deuteration and Resonance Raman Spectroscopic Characterization of Biliverdin and Phycocyanobilin. *Chem. - A Eur. J.* **3**, 363–367 (1997).
2. F. Celis, *et al.*, Surface-enhanced Raman scattering and theoretical study of the bilichromes biliverdin and bilirubin. *Spectrosc. Lett.* **49**, 336–342 (2016).
